# What Cocaine to Use

**Published:** 1888-09

**Authors:** 


What Cocaine to Use.—There are many brands of cocaine in the market, and many physicians have found to their annoyance that some are inert and some very irritating when applied to a sensitive membrane.

It may, therefore, be of service to physicians to learn the experience of Dr. Dudley S. Reynolds, editor of *Progress*, who in the July, 1888, number expresses himself as follows:

"The medical profession has about settled its estimate of the therapeutical value of muriate of cocaine, but it is, unhappily, no easy matter to decide upon the most uniformly reliable source of supply. The editor of *Progress* had about concluded Merck's was the only reliable product, when recently he was induced to make trial of that produced by Parke, Davis & Co. A fresh sample of ten grains was dissolved in five drachms of distilled water, to which was added one drop of liquid carbolic acid. One drop of this instilled into the eye of a man from whose cornea a foreign body was to be removed, produced complete anæsthesia in three minutes, so that incision of the inflamed cornea, and turning out of the piece of offending metal was not felt by the patient. Twenty other similar experiments yielded similar results."

